# Damage Detection of a Concrete Column Subject to Blast Loads Using Embedded Piezoceramic Transducers

**DOI:** 10.3390/s18051377

**Published:** 2018-04-28

**Authors:** Kai Xu, Qingshan Deng, Lujun Cai, Siuchun Ho, Gangbing Song

**Affiliations:** 1 College of Urban Construction, Wuhan University of Science and Technology, Wuhan 430065, China; xukai@wust.edu.cn (K.X.); shanadolph@163.com (Q.D.); 2 College of Science, Wuhan University of Science and Technology, Wuhan 430065, China; 3 Department of Mechanical Engineering, University of Houston, Houston, TX 77204, USA; smho@uh.edu

**Keywords:** blast loads, damage detection, piezoceramic transducers, smart aggregate (SA), concrete column, wavelet packet analysis, damage index

## Abstract

Some of the most severe structural loadings come in the form of blast loads, which may be caused by severe accidents or even terrorist activities. Most commonly after exposure to explosive forces, a structure will suffer from different degrees of damage, and even progress towards a state of collapse. Therefore, damage detection of a structure subject to explosive loads is of importance. This paper proposes a new approach to damage detection of a concrete column structure subjected to blast loads using embedded piezoceramic smart aggregates (SAs). Since the sensors are embedded in the structure, the proposed active-sensing based approach is more sensitive to internal or through cracks than surface damage. In the active sensing approach, the embedded SAs act as actuators and sensors, that can respectively generate and detect stress waves. If the stress wave propagates across a crack, the energy of the wave attenuates, and the reduction of the energy compared to the healthy baseline is indicative of a damage. With a damage index matrix constructed by signals obtained from an array of SAs, cracks caused by blast loads can be detected throughout the structure. Conventional sensing methods such as the measurement of dynamic strain and acceleration were included in the experiment. Since columns are critical elements needed to prevent structural collapse, knowledge of their integrity and damage conditions is essential for safety after exposure to blast loads. In this research, a concrete column with embedded SAs was chosen as the specimen, and a series of explosive tests were conducted on the column. Experimental results reveal that surface damages, though appear severe, cause minor changes in the damage index, and through cracks result in significant increase of the damage index, demonstrating the effectiveness of the active sensing, enabled by embedded SAs, in damage monitoring of the column under blast loads, and thus providing a reliable indication of structural integrity in the event of blast loads.

## 1. Introduction

Recent years have seen a rise in terrorist activities, and the number of bombing incidents in particular have risen across the globe. Terrorists have been known to indiscriminately target civilian populations, thus exposing civil structures to dangerous blast loads [1,2]. Furthermore, with the use of vehicles and suitcase bombs against critical elements in city centers, the potential for greatly increased blast magnitudes have intensified [3,4]. An explosion with adequate destructive force denoted within or near a building can cause catastrophic damage to the building’s external and internal structures, leading to immediate or progressive collapse [5].

Progressive collapse is a chain reaction of failures initiated by instantaneous loss of at least one load carrying element [6]. Such a mode of structural failure occurred during several actual explosion events [7,8,9,10,11]. Much research has been conducted on the assessment of impact and risk analysis of the entire structure in which blasting loads destroyed critical columns. Izzuddin et al. [12] and Vlassis et al. [13] presented a simplified, design-oriented method for assessing the progressive collapse of multi-story buildings subject to sudden loss of a peripheral or a corner column. Sasani [14] evaluated the response of Hotel San Diego, a six-story reinforced concrete frame structure that suffered from the simultaneous removal of two adjacent exterior columns. Parisi [15] derived a multi-level, pressure-impulse diagram which quantifies the loss of axial load-bearing capacity experienced by the reinforced concrete (RC) column. Hao et al. [16] and Grant and Stewart [17] developed a probabilistic risk-assessment model for the failure probabilities of RC columns subjected to blast loads. All these evaluation methods to assess the risk of collapse are based on the losses or damages of the columns in the structure; however, due to the dynamic complexity of blast loads, it is not easy to determine damage severity to include information about invalidated columns. Therefore, the ability to accurately and rapidly detect and quantify the damage to columns subjected to extreme loads will be of great importance for assessment of structural viability after an attack. For RC structures, there will be an additional emphasis on the detection of the cracks in the structure.

Various non-destructive testing (NDT) techniques are available to detect the condition of critical elements in a structure. Among the most popular NDT techniques used in blast engineering are the ultrasonic velocity method, ground penetrating radar (GRP) techniques, impact-echo (IE) approaches, among others [18]. Štoller and Zezulová [19] used the ultrasonic velocity method for the diagnosis of protective RC-structure elements under blast loads. Lai et al. [20] adopted ultrasonic velocity method to detect the damage of the ultra-high performance concrete under repeated penetration and explosions at different depths. Sham and Lai [21] utilized the GRP technique for the detection of cracks and prediction of damages in actual concrete structures. Epasto et al. [22] applied the impact-echo method for fire-damage detection on concrete using a wavelet time-frequency technique.

In recent years, piezoceramic materials, such as lead zirconate titanate (PZT), have been widely used in structural health monitoring (SHM) [23,24,25,26,27] and damage detection [28,29,30], due to their low cost, strong piezoelectric effect [31], broadband frequency response [32], the ability to be used as actuators [33,34] and sensors [35], and the capacity to harvest energy [36]. Furthermore, PZT can be inexpensively formed into different geometries to meet specific applications [37,38]. Additionally, the piezoceramic-based active sensing method has been accepted in the field of SHM due to its effectiveness and reliability, including usage for diagnosing the damage of structure in blast engineering [39]. However, PZT is fragile and use in harsh environments require additional protection. For embedment into concrete structures, the smart aggregate (SA) was fabricated by sandwiching a waterproofed PZT patch between two concrete blocks [40]. The proposed smart aggregate is multi-functional and has enabled a number of damage detection and monitoring work [41]. Liao et al. [42] utilized SAs to monitor the damage of an RC column under earthquake loading. Laskar et al. [43] evaluated the health status of a two-story RC frame instrumented with SAs. The frame was subjected to a monotonic lateral load up to failure, and the SAs captured the evolution of structural damage during the process. Zou et al. [44] and Liu et al. [45] studied concrete water seepage monitoring using smart aggregates. Gu et al. [46] tested two columns subjected to seismic excitations. In the test, the SAs were used to perform multiple monitoring functions including dynamic seismic response detection and structural health monitoring. Using SA transducers, Kong et al. [47] studied the cracking of an RC bridge column subjected to pseudo-dynamic loadings. Thus smart aggregates can be embedded into the structure and provide comprehensive monitoring of concrete structures throughout their lifetime. However, so far, SAs have yet to be utilized in the damage detection of structures under blast loads.

Since columns are critical elements needed to prevent structural collapse, knowledge of their integrity and damage conditions is essential for safety after exposure to blast loads. Without accurate information about column health, any risk evaluations of the entire structure may not be reliable. In this research, a new approach to damage detection of a concrete column structure subject blast loads using embedded piezoceramic-based devices, smart aggregates (SAs), is proposed. Since the sensors are embedded in the structure, the proposed active-sensing based approach is more sensitive to internal or through cracks than the surface damage. To facilitate the proposed research, a concrete column specimen was fabricated and subjected to air-blast loadings in an explosion containment vessel. The column was placed in a horizontal position with no axial load, and one end of the column was fixed into a concrete bed while the other end was supported by a steel roller to simulate the inflection point (i.e., the actual deformation of a column in the frame structure). Five blasting tests were implemented at different standoff distances above the column. Since the mass of the charges were controlled to a very low level, it is possible to detect the development of the cracks and evaluate the accumulation of damage in the column. Four SAs were installed in the column prior to pouring, two of which were used as actuators to generate probing stress waves, while the other two were used as sensors to detect the stress waves. Blast loads applied on the column caused a damage zone between the actuators and sensors that would directly influence the stress wave attenuation through this area. The stress wave energy attenuated more when travelling through a cracked area. In this paper, the changes of received signal energy were analyzed by wavelet packet analysis, and a damage index was developed to quantify the damage status in the column. For the measurement purpose, dynamic strain gauges and an accelerometer were installed on the column specimen. Experimental results reveal that surface damages, though appear severe, cause minor changes in the damage index, and through cracks result in significant increase of the damage index, demonstrating the effectiveness of the active sensing, enabled by embedded SAs, in damage monitoring of the column under blast loads.

## 2. Smart Aggregate-Based Active Sensing Approach

### 2.1. Smart Aggregates

**Figure 1:**
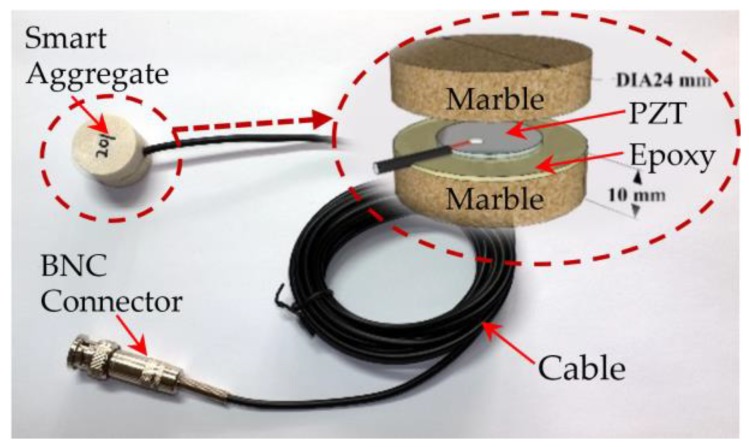
Illustration of a smart aggregate (SA).

Piezoceramic materials are brittle and cannot be directly used without protection, therefore a smart aggregate (SA) is fabricated to protect the PZT disk by sandwiching it between two marble blocks with epoxy, as shown in Figure 1. The diameter of the PZT disk is 15 mm, and the thickness is 0.3 mm. With the protection, the PZT transducer can be used in harsh environments. A BNC connector with a cable is used to offer an electric connection to the smart aggregate.

### 2.2. Damage Detection Principle

Essentially, an SA is a kind of protected piezoelectric transducer. It will generate an electric charge when subjected to a stress or strain, which is called the direct piezoelectric effect; conversely, it also can produce stress or strain when an electric field is applied to it, which is called the inverse piezoelectric effect [48]. 

**Figure 2:**
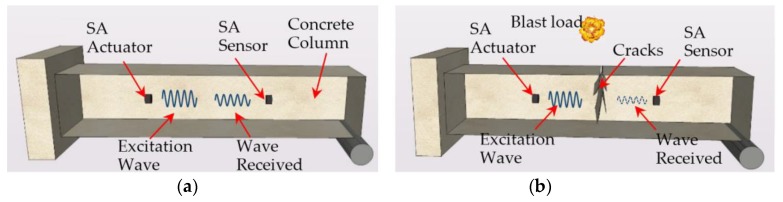
Smart aggregate-based active sensing approach to detect damage of concrete structure: (**a**) The stress wave propagation through the structure without damage (before an explosion); (**b**) The stress wave propagation through the structure with a damage (after an explosion).

Figure 2 illustrates the principle of a smart aggregate-based active sensing system for the damage monitoring of a concrete column after an explosive event. As shown in Figure 2, there is a pair of SAs embedded in the concrete column. One of them is used as an actuator to generate stress a wave that will propagate along the structure, while the other SA is used as a sensor to detect the stress wave. As shown in Figure 2b, a crack in the concrete structure acts as a relief for the stress wave along the propagation path, which significantly attenuates the energy that the stress wave carries. Therefore, by analyzing the detected stress wave, the degree of the damage of the concrete structure can be monitored. Since the sensors are embedded, the proposed active sensing based approach is more sensitive to internal or through cracks than the surface cracks.

### 2.3. Damage Index and Damage Index Matrices

Wavelet packet analysis enables the inspection of a signal in relatively narrow frequency bands over a relatively short time window. It is an effective signal-processing tool for damage feature extraction. In this paper, wavelet packet-based analysis is applied to offer the corresponding values of the received energy due to different damages. The sensor signal can be decomposed by an *n*-level wavelet packet into 2*^n^* signal sets $\left\{X_{1},X_{2},\cdots ,X_{2^{n}}\right\}$ [49], where *X_j_* can be expressed as:

(1) $$X_{j}=\left[x_{j,\;1},x_{j,\;2},\cdots ,x_{j,m}\right]$$

where *m* is the number of the sampled data. Additionally, the energy of the decomposed signal is defined as:

(2) $$E_{i,j}={\left\|X_{j}\right\|}_{}^{2}=x_{j,\;1}^{2}+x_{j,\;2}^{2}+\cdots +x_{j,\;m}^{2}$$

where *i* is the time index, and *j* is the frequency band number (*j* = 1 … 2*^n^*). The proposed blast damaged index at the *i*th time is defined as:

(3) $$I=\sqrt{\sum_{j=1}^{2^{n}}{\left(E_{i,\;j}-E_{h,j}\right)}^{2}/\sum_{j=1}^{2^{n}}E_{h,j}{}^{2}}$$

where *E_h, j_* is the energy of the decomposed sensor signal at the *j*th frequency band in the healthy status.

The blast damage index represents the loss portion of the transmission energy. When the index is close to zero, the structure is in a healthy status. When the damage index goes up to a certain threshold value, that means the severe damage occurs in the structure. In this case, the higher the index, the severer the damage. To demonstrate the health status at different locations of the concrete structure, a Sensor-History Damage Index Matrix (SHDIM) **M***_r×s_* [50] is defined as:

(4) $$M_{r\times s}={\left|I_{p,\;q}\right|}_{r\times s},\;\;\;\;p=1,\cdots r\;\;and\;\;q=1,\cdots ,s$$

where the matrix element at the *p*th row and the *q*th column, *I_p_*_,*q*_ is the damage index of the *p*th smart aggregate at the time of the *q*th test (i.e., *p* is the sensor index, *q* is the time index); *r* is the total number of smart aggregates and *s* is the total number of tests. The damage status at different positions of the specimen at different test times can be described by a 3-dimensional damage index matrix plot. 

## 3. Specimen and Experimental Setup

### 3.1. Test Specimen

**Figure 3:**
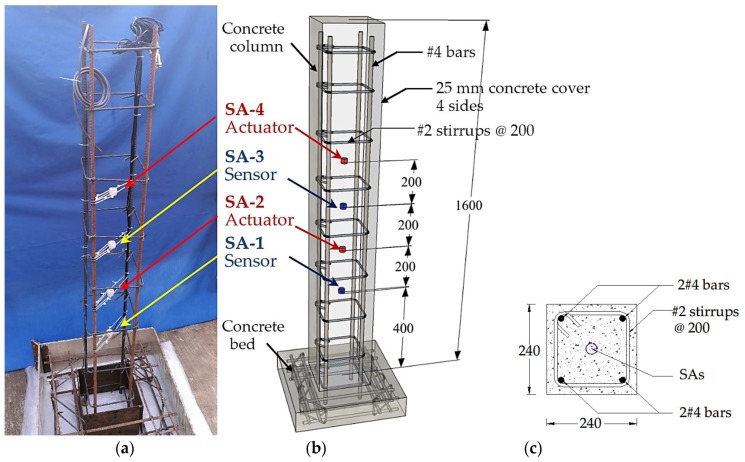
Location of smart aggregates (all dimensions are in mm): (**a**) A photo of reinforcing cage; (**b**) Fabrication of the column; (**c**) A cross-sectional view of the column.

In this research, a concrete column, as shown in Figure 3, is designed and fabricated as the test specimen. For the column, the height is 1.6 m or 1600 mm, and its cross-section is 240 mm × 240 mm. Other dimensions are shown in Figure 3. The detailed concrete mix proportion is shown in Table 1. As shown in Figure 3, four smart aggregates were installed at the pre-determined locations along the steel rebars with the help of plastic zip ties before pouring the concrete. The detailed location of each SA is shown in Figure 3b. The four SAs are numbered 1–4. Please note that SA2 and SA4 are the actuators and SA1 and SA3 are the sensors. The specimen was cured for 28 days following the standard curing procedure. The ASTM has designated five types of Portland cement, of which the one used was designated as Type I. 

**Figure 4:**
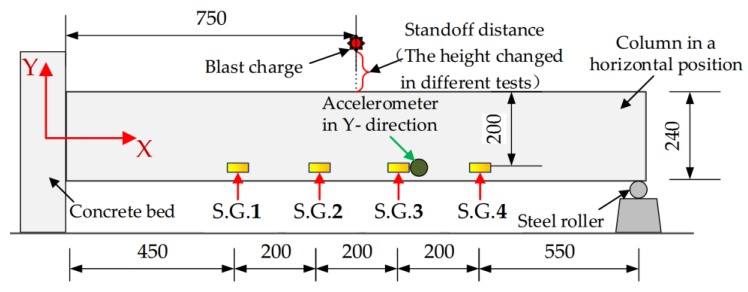
Location details of the explosive charge, strain gauges (S.G.) and accelerometer (units: mm).

In addition, as shown in Figure 4, which depicts the column in the horizontal position, four strain gauges (S.G.) were surface-bonded on the specimen. The four strain gauges, whose detailed locations are also shown in Figure 4, are numbered S.G. 1 through S.G.4. An accelerometer was installed next to S.G.3 and was used to capture the acceleration of the specimen. Figure 4 also shows the placement of the explosive charge suspended above the column.

### 3.2. The Experimental Setup

**Figure 5:**
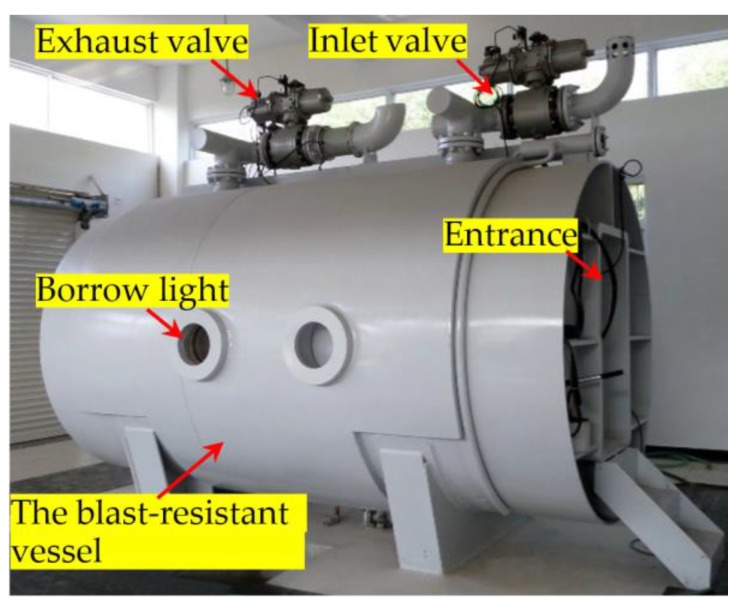
A photo of the Explosion Containment Vessel.

**Figure 6:**
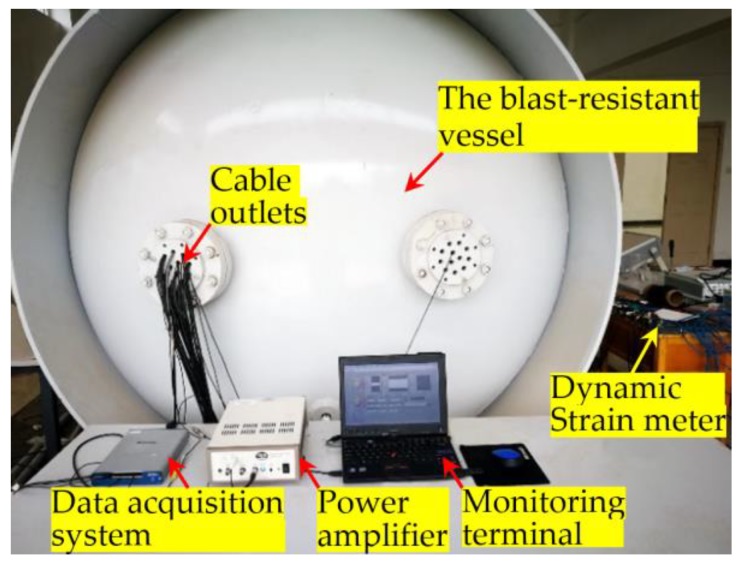
The instrumentation setup.

**Figure 7:**
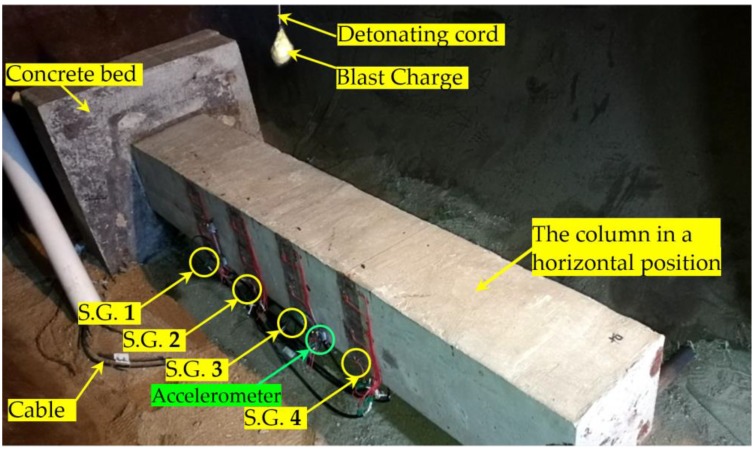
A photo of the specimen in the EVC (before the explosion).

In this experimental study, a thick-walled explosion containment vessel (ECV), as shown in Figure 5, was used to host the specimen during an explosion. Figure 6 shows the instrumentation setup for the experiments. Figure 7 shows the specimen in the ECV.

The SAs were controlled and monitored through a multifunction data acquisition and control system (DACS). The SAs as sensors are connected to the A/D (analog to digital) convertor of DACS. The SA as an actuator is connected to the D/A (digital to analog) convertor of the DACS through a power amplifier. A laptop PC was used to interface with the system and record the data. During the tests, the pre-amplified excitation voltage is 3V for the D/A convertor. A swept sine wave was employed to excite the SAs repeatedly. The sinusoidal sweep signal increased linearly from 100 Hz to 200 kHz in a one-second cycle, and the sampling frequency was 2 MS/s for the data acquisition and control system. The amplifier provided a fixed gain of 10×, and an operation bandwidth of 0.1–200 kHz.

For comparison, a dynamic strain meter was used to record vertical acceleration and the dynamic strain in the axial direction. The sampling frequency of the strain gage was 128 kHz. 

### 3.3. The Experimental Procedure

As shown in Figure 6, the blast charge is placed above the specimen, and the detailed location of the explosive is shown in Figure 4. During the tests, a total of five explosions were used. Table 2 shows the details of each explosion. 

Test No. B0 is used to represent the healthy status and B1–B5 represent the five different explosions. Before the first explosion and after each explosion, smart aggregate based active sensing was conducted following the actuator-sensor sequences, as shown in Table 3. For example, the Signal No. P1 represents the signal detected by SA-3 when it is used as a sensor while SA-4 is used as the actuator. For Tests B1–B4, as listed in Table 2, the blast charges were placed away from the structure. However, the distance between the charges and the structure became less with the procedure of the experiments. Please note that, for Test No. B5, the blast charge was directly placed on the surface of the concrete structure, and this blast will cause the most severe damage to the structure. To detect the development of the cracks and assess the accumulation of damage in the column by each blast load, the mass of each blast charge was 40 g, which is at a relatively low level. The term “charge” here means “TNT equivalent of the shock wave”.

## 4. Experimental Results and Discussion

### 4.1. The Results of Strain and Acceleration

**Figure 8:**
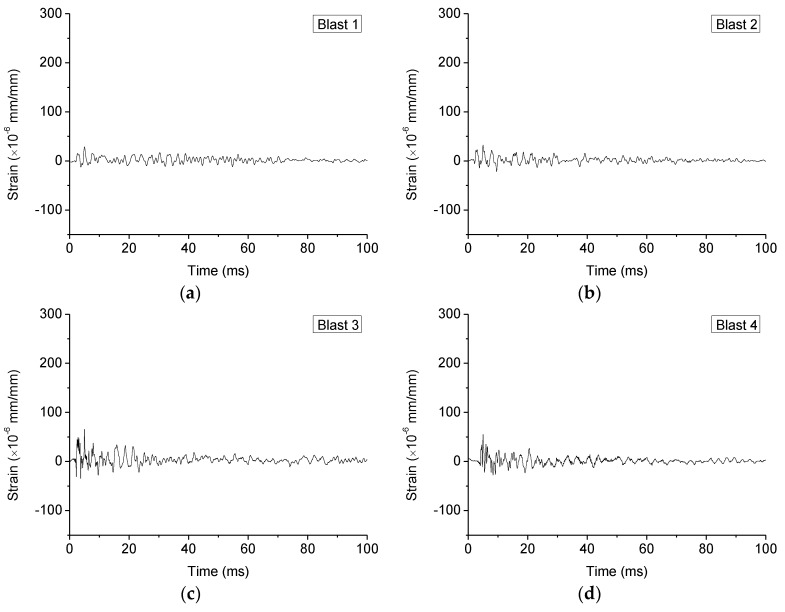

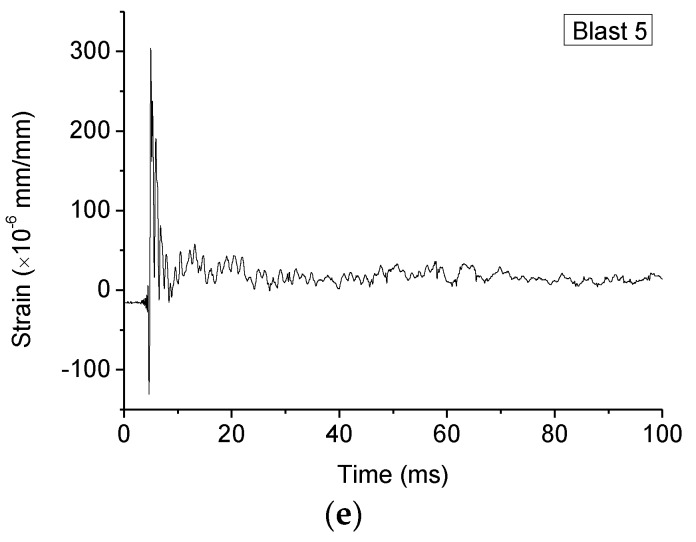
Time responses of a strain gauge (**S.G.2**): (**a**) under 1st blast loading; (**b**) under 2nd blast loading; (**c**) under 3rd blast loading; (**d**) under 4th blast loading; (**e**) under 5th blast loading.

**Figure 9:**
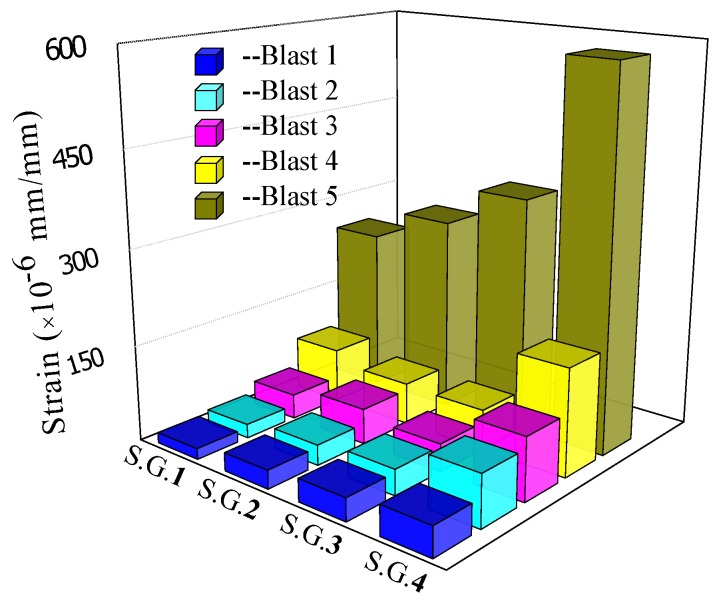
The maximum strain of each gauge after different blast loadings.

Figure 8 plots the time responses of the 2nd strain gauge (S.G. 2) during each blast. Figure 9 shows the maximum response of all the strain gauges during all blasts. 

**Figure 10:**
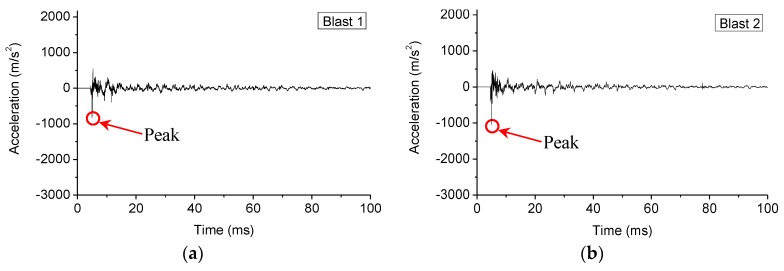

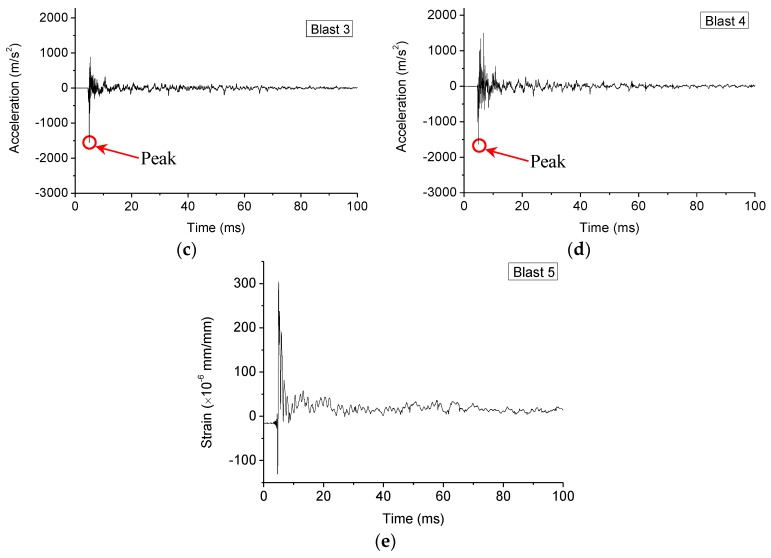
Time responses of the accelerometer (in 100 ms): (**a**) under 1st blast loading; (**b**) under 2nd blast loading; (**c**) under 3rd blast loading; (**d**) under 4th blast loading; (**e**) under 5th blast loading.

**Figure 11:**
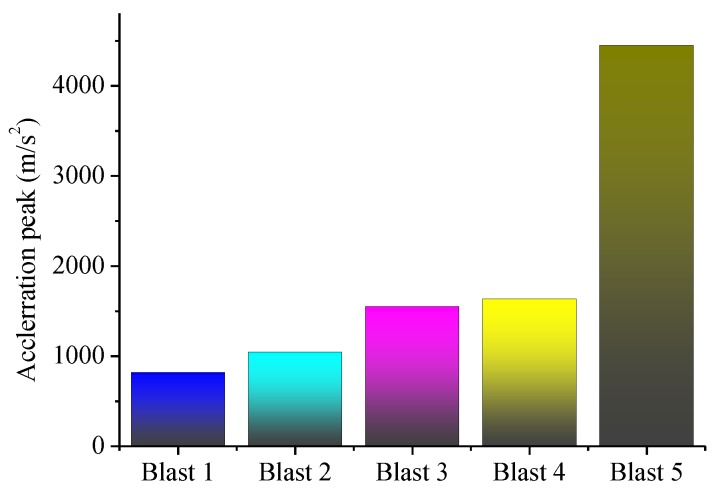
The acceleration peaks of each blast loading (absolute values).

It is clear that the maximum strain grows gradually with the decrease of standoff distances, and the last blast resulted in the most strain since the charge was directly placed on the specimen. This phenomenon is also reflected in the acceleration responses of the specimen, as shown in Figure 10, which plots the time responses of the acceleration during each blast, and Figure 11, which depicts the maximum acceleration during each blast. Please note that, to ensure to acquire the needed data, the data acquisition system was turned on 5 ms prior to the detonation. 

As clearly shown in Figure 10, the peak of the time history curve that corresponds to the blast is at 5 ms. Moreover, in Figure 9, the maximum strains from different gauges increased from S.G. 1 to S.G. 4, which are near to the fixed end and the simply supported end, respectively. This phenomenon shows that the different constraint conditions will influence the deformation of the structure under blast loads.

### 4.2. The Results of Piezoelectric Active Sensing

**Figure 12:**
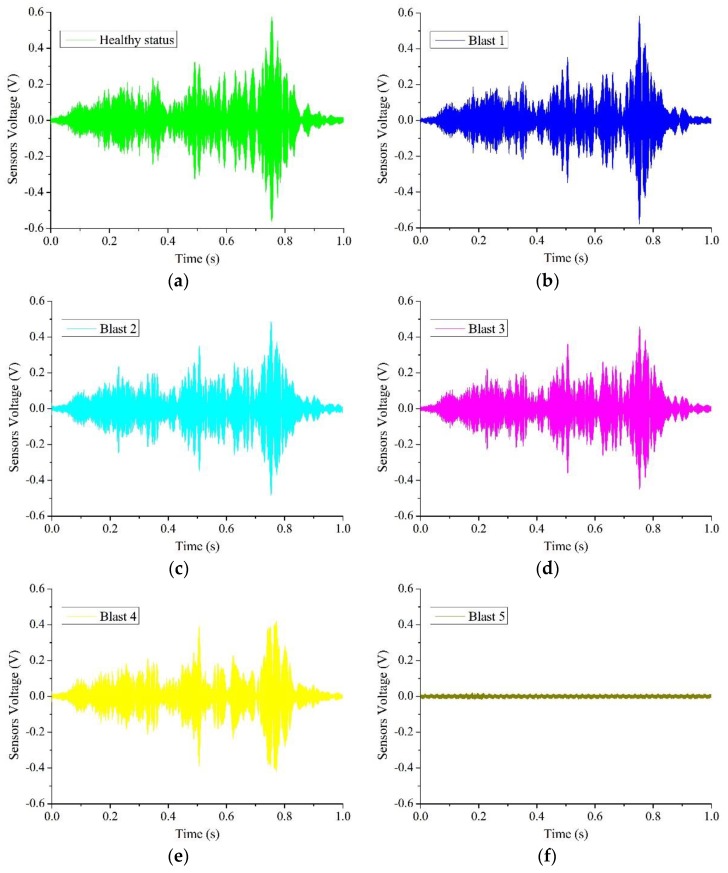
Time responses of a smart aggregate (Signal **No. P2**): (**a**) healthy status; (**b**) after 1st blast loading; (**c**) after 2nd blast loading; (**d**) after 3rd blast loading; (**e**) after 4th blast loading; (**f**) after 5th blast loading.

**Figure 13:**
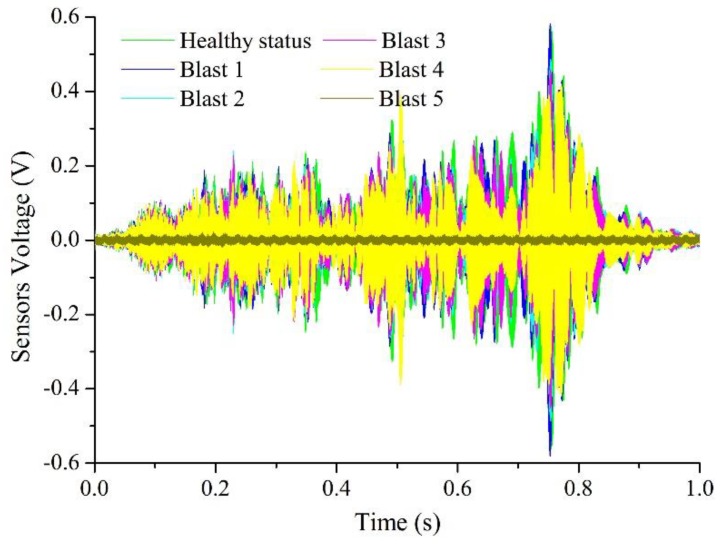
Time responses of Signal **P2** after different blast loadings (composite graph).

**Figure 14:**
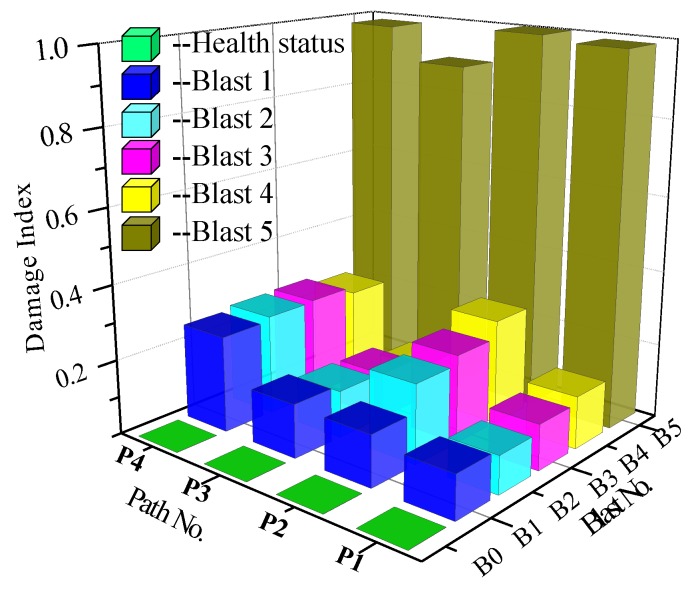
The damage index matrix.

The results of the smart aggregate based active sensing of the signal path P2 (SA-2 as an actuator and SA-3 as a sensor) for the five blast are shown in Figure 12a–e. For an easy comparison, the overlapping plots of the signals in Figure 12a–f are shown in Figure 13. It is clear that at the healthy status, the sensor signal has a large magnitude. The magnitude decreases with the increase of blast number gradually up to Blast 4. For Blast 5, the charge was directly placed on the specimen. After the last blast, the magnitude of the signal significantly dropped and was almost zero, which indicates a severe damage to the structure and the stress wave can no longer propagate from the actuator to the sensor. This trend is also verified by other signal paths, as shown in the damage index matrix (Figure 14), which clearly shows the significant increases of the damage index values for all signals after the last blast. 

**Figure 15:**
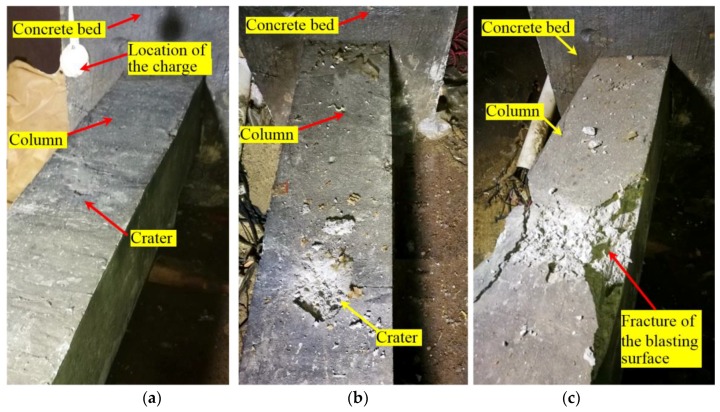
The damage situations after explosions: (**a**) After Blast 2; (**b**) After Blast 4; (**c**) After Blast 5.

**Figure 16:**
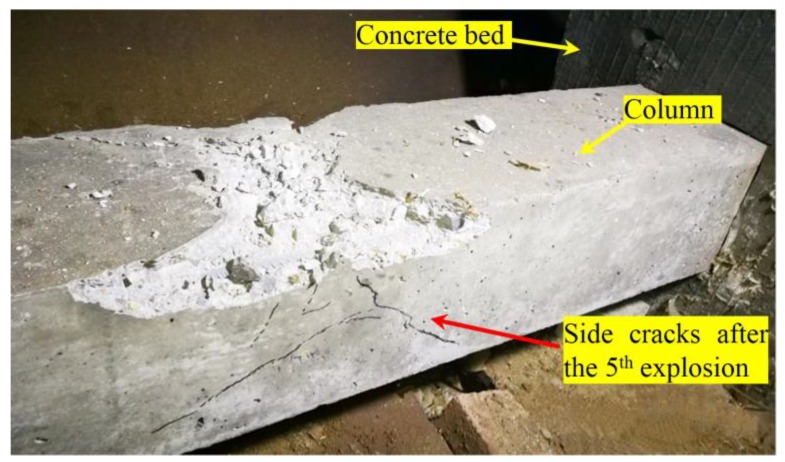
The column specimen after Blast 5: the through cracks are clearly visible.

All these indicate that the structure was severely damaged after the last blast, which is verified by the damages to structure graphically (Figure 15 and Figure 16). Figure 15a shows very minor surface damages after the first two blasts. Figure 15b shows the column after the Blast 4 and we can see severe surface damages (a large but not-deep crater and cracks). After a careful check, these cracks remain at the surface and no through crack was observed. For Figure 15b, the damages appears severe, however, only remain at the surface, which is consistent with the fact all the actuator-sensor signals after Blast 4 are still strong. From these photos, the blast prior to the last one caused no or minor damages (superficial or surface damages and cracks) to the structure (Figure 15a). As shown in Figure 15c and further verified in Figure 16, the last blast indeed resulted in significant damages (through cracks) to the structure, which was detected by the smart aggregate based active sensing.

## 5. Discussion

Concrete is a kind of material that possesses initial micro-cracks. These initial cracks will extend and join each other when disturbed by a severe impact load, and the joining of the cracks results in a certain degree of damage to the structure. The merging of the micro-cracks after the first blast (B1) can be indicated with the blast damage index, as shown in Figure 14. Further cracking requires a considerable amount of additional energy. However, as shown in Figure 9, under the blast loads B2-B4, the maximum strain at the monitoring points have yet to reach their ultimate value, which means according to the strain gauges, the blast loads were not enough to trigger severe damage to the specimen. This conclusion can also be drawn from the SA data in Figure 14, in which the damage index does not rise sharply after the blast loads B2–B4. In addition, the observation of the craters on the structure, as shown in Figure 15b, does not necessarily accurately indicate the true damage severity of the structure. Since the smart aggregates were embedded in the concrete column, the stress waves propagate inside the column, and the damage index value reflect the internal damages to the structure. Corresponding the case of Figure 15b, after Blast 4.

The final blast, which exploded directly on the surface of the structure, severely damaged the structure. As shown in Figure 15, penetration or through cracks were evident in the structure, and the corresponding blast damage index is close to 1. An index of 1 means the structure is 100% damaged at the moment. The propagating path P2 of the stress wave was immediately below the explosive charge. Therefore, the column damage reflected by the signal of P2 in each explosion is higher than those of the P1 and P3, which were further away than P2. The monitoring signal P4 detected structural damage at a longer distance. It can be seen from Figure 13 that P4 reflects an accumulation of damages from several other paths. In summary, since the sensors are embedded, the proposed active-sensing based approach can clearly detect the through cracks as a result of blast damages.

## 6. Conclusions and Future Work

In this paper, the damage status of a concrete column under blast loads is monitored by an active sensing method enabled by embedded piezoceramic smart aggregate (SA) transducers. Since the cracks caused by blast loads weaken the stress wave propagation energy across the detection area, the amplitude of the time-domain signal received by embedded PZT sensors decreases when the cracks occur. A wavelet packet-based blast damage index is used to report the initiation and the further evolution of the damage status. Since the sensors are embedded in the structure, the proposed active-sensing based approach is more sensitive to internal or through cracks than surface damages. In addition, the values in damage index matric obtained by distributed PZT sensors not only describe the damage evolution process but also reflect the local severity of damage condition in the concrete structure. Experimental results reveal that surface damages, though appear severe, cause minor changes in the damage index, and through cracks, which dramatically weaken a structure, result in significant increase of the damage index, demonstrating the effectiveness of the proposed active sensing method, enabled by embedded SAs, in damage monitoring of the column under blast loads, and thus providing a reliable indication of structural integrity in the event of blast loads. Future work will include more systematic studies of monitoring of the damages on a concrete structure subject to blast loads using SAs. One of the test will involve investigating the damage index increase when the blast loads are repeated at the same distance from the structure. 

## Figures and Tables

**Table 1:** Detailed concrete mix composition.

Component	Quantity (Kg/m^3^)	Description
Cement	500	Portland cement (Type I)
Sand	479	Standard sand
Aggregate	1231	16 mm size angular limestone
Water	190	Water

**Table 2:** Experimental program.

Test No.	TNT Charge Mass	Standoff Distance	Description
B0	-	-	Healthy status
B1	40 g	50 cm	Blast 1
B2	40 g	40 cm	Blast 2
B3	40 g	30 cm	Blast 3
B4	40 g	20 cm	Blast 4
B5	40 g	0 cm	Blast 5

**Table 3:** Actuator-Sensor Sequence.

Propagation Path No.	Schematic	Propagation Direction	Propagation Distance
P1	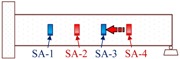	From SA-4 to SA-3	20 cm
P2	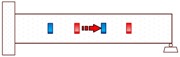	From SA-2 to SA-3	20 cm
P3	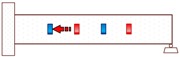	From SA-2 to SA-1	20 cm
P4	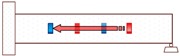	From SA-4 to SA-1	60 cm
